# Phenotypic characterization and antimicrobial resistance of *Campylobacter jejuni* and *Campylobacter coli* in broiler chickens and slaughterhouse workers in Algeria

**DOI:** 10.5455/javar.2025.l952

**Published:** 2025-09-22

**Authors:** Hakima Messaoudene, Meryem Guessoum, Nouzha Heleili, Ammar Ayachi

**Affiliations:** 1 Laboratory of the Environment, Health and Animal Production, Institute of Veterinary and Agricultural Sciences, University of Batna 1, Batna, Algeria; 2 Local Animal Resources Management Laboratory, National Veterinary School, El-Harrach, Algeria; 3 Laboratory of Biotechnology, Bioactive Molecules and Cellular Pathophysiology, University of Batna 2, Batna, Algeria

**Keywords:** Antimicrobial resistance, multidrug resistance, one health, poultry, Slaughterhouse workers

## Abstract

**Objectives:**

This study assessed the prevalence, phenotypic antimicrobial resistance profiles, and multidrug resistance patterns of* Campylobacter jejuni* (*C*. *jejuni*) and *Campylobacter coli* (*C*. *coli*) isolated from broiler chickens and slaughterhouse workers in Algeria to craft One Health strategies.

**Material and Methods:**

Samples were collected from poultry carcasses (*n =* 300) and slaughterhouse workers (*n =* 133). *Campylobacter *spp. were isolated and identified using classical phenotypic microbiological methods, followed by antimicrobial susceptibility testing to assess resistance patterns, including MDR profiles.

**Results:**

*Campylobacter* spp. were detected in 66.3% of samples, with the highest prevalence in caeca (96%) and cloacal swabs (70%), while lower rates were observed in neck skin (33%). Among slaughterhouse workers, *Campylobacter* was detected in 3.9% of samples from workers in semi-industrial settings and in 5.9% of hand scrapings. *Campylobacter* *jejuni* was the predominant species (29%), followed by *C*. *coli* (11.3%). High resistance rates were observed against ciprofloxacin (82.6% in *C*. *jejuni*, 58% in *C*. *coli*) and tetracycline (48% in *C*. *jejuni*). Workers’ isolates exhibited 100% resistance to ampicillin, with moderate resistance to other antibiotics. Multidrug resistance was most frequently observed in *C*. *jejuni*, particularly against ciprofloxacin, tetracycline, and erythromycin.

**Conclusion:**

These findings underscore poultry as critical reservoirs of thermotolerant *Campylobacter *and the urgent need for a coordinated One Health approach, which is vital to minimize the risk of bacterial transmission and *Campylobacter* infections, as well as to combat antibiotic resistance. This approach integrates surveillance and control measures to address the interconnection between human, animal, and environmental health in Algeria.

## Introduction

*Campylobacter* spp. are Gram-negative, spiral-shaped, microaerophilic bacteria equipped with polar flagella, enabling motility in viscous environments [1]. Among the species, *Campylobacter jejuni* (*C*. *jejuni*) and *Campylobacter coli* (*C*. *coli*) are thermotolerant species that grow optimally at 42°C, reflecting their habitats in the avian cecum [2]. These pathogens possess various virulence mechanisms, including adhesion, invasion, and toxin production, which facilitate evasion of host defenses and play a role in pathogenicity [2,3]. Although *Campylobacter* species are considered commensals in the intestinal tract of poultry, they are major zoonotic agents responsible for human campylobacteriosis, one of the most common causes of bacterial gastroenteritis in developed countries [4–6].

Human infection occurs primarily through the consumption of raw or undercooked poultry meat, contaminated water and milk, and direct contact with animals [3,4,7]. Poultry carcasses often become contaminated during slaughterhouse processing, with varying contamination levels based on facility types (e.g., small-scale *vs.* industrial operations) [8-10]. Campylobacteriosis generally presents with clinical manifestations that include gastrointestinal symptoms, mainly diarrhea, and has been associated with a range of conditions such as inflammatory bowel disease, esophageal disease, functional gastrointestinal disorders, celiac disease, and cholecystitis [11,12]. In severe cases, infections may progress to systemic complications such as Guillain-Barré syndrome, reactive arthritis, or bacteremia, necessitating antibiotic therapy [13-15]. However, the increasing prevalence of antimicrobial resistance (AMR) in *Campylobacter *spp., driven by the misuse of antibiotics in livestock production, complicates treatment and poses a growing global public health crisis [16,17].

*Campylobacter* *jejuni* and *C*. *coli* commonly exhibit resistance to critically important antibiotics, including macrolides (e.g., erythromycin), fluoroquinolones (e.g., ciprofloxacin), tetracyclines, and β-lactams [16,18,19]. Resistance mechanisms may be intrinsic, such as efflux pumps or mutations in target genes, or acquired via horizontal gene transfer, further exacerbating the spread of multidrug-resistant (MDR) strains [20–22]. In North Africa, including Algeria, studies have documented high prevalence rates of *Campylobacter* in poultry and humans, along with alarming trends in AMR [20]. Despite these findings, critical knowledge gaps remain, particularly concerning occupational exposure risks among slaughterhouse workers and region-specific AMR profiles in eastern Algeria.

This study aims to address these gaps by investigating the prevalence and phenotypic AMR patterns of *C*. *jejuni* and *C*. *coli* in broiler chickens and slaughterhouse workers in Algeria. Specifically, it focuses on three key objectives: [1] to assess contamination levels across broiler chicken samples (cloacal swabs, caeca, and neck skin), slaughterhouse surfaces, and workers’ hands; [2] to characterize AMR and MDR profiles to identify high-risk resistance combinations; and [3] to provide baseline data to support One Health strategies for reducing zoonotic transmission and guiding antibiotic stewardship in poultry production.

## Materials and Methods

### Ethical approval

This study received ethical approval from the Scientific Committee of the Institute of Veterinary and Agricultural Sciences, University of Batna 1, Algeria, under reference number N°/DV/ISVSA/UB1/2024, issued on January 20, 2025.

### Study design 

The study was conducted across three poultry slaughterhouses located in Batna Province, northeastern Algeria (435 km southeast of Algiers), selected to represent varying operational scales: (A) two industrial slaughterhouses, modern facilities with large-scale poultry rearing and processing in the East and South regions, and (B) a semi-industrial slaughterhouse with limited resources and serving local markets. These slaughterhouses were chosen based on their operational availability during the sampling period [from September 2023 to August 2024].

### Sample collection

A total of 433 samples were collected randomly, including 300 poultry samples (100 cloacal swabs, 100 cecal contents, and 100 neck skin) and 133 human samples from slaughterhouse workers (73 from semi-industrial and 60 from industrial facilities). Approximately 30 samples were collected during each of the 10 visits. There were two batches per visit. This was done to ensure the diversity and representativeness of the batches of chickens sampled. Some samples were also collected from the staff at the same time. Poultry samples were collected aseptically: cloacal swabs (pre-evisceration) using sterile cotton swabs; cecal contents (post-evisceration) into sterile screw-cap containers; and neck skin excised using sterile scalpels. Worker samples included hand scrapings (aseptically collected from fingernails and interdigital spaces) and fecal samples (collected directly into sterile jars). All samples were labeled (date and subject or batch ID) and then transported on ice to the Microbiology Laboratory at the University of Batna 1 within 3–4 h of collection.

### Isolation and identification of Campylobacter jejuni and C. coli

Isolation followed ISO-10272 protocols [23,24]. Swabs from cloacal, cecum, and human sources were streaked directly onto modified Charcoal Cefoperazone Desoxycholate Agar (mCCDA; Oxoid, France) supplemented with *Campylobacter* growth supplement, modified (SR155H), and Blaser-Wang selective supplement (SR83, Oxoid). Neck skin samples were homogenized in Bolton.

Broth (Oxoid) supplemented with 5% horse blood and incubated microaerophilically (5% O₂, 10% CO₂, and 85% N₂) at 42°C for 48 h before sub-culturing onto mCCDA plates. Presumptive *Campylobacter* colonies (grey, moist, and spreading morphology) were further analyzed with Gram staining (spiral and Gram-negative rods), corkscrew motility under phase-contrast microscopy, and biochemical assays (oxidase, catalase activity, Hippurate hydrolysis, and H₂S production on triple sugar iron agar). Species differentiation relied on Cephalothin resistance (30 µg) and Nalidixic acid susceptibility (30 µg). Control strains (*C*. *jejuni* ATCC 33560 and *C*.* coli* ATCC 33559) were also used.

### Antimicrobial susceptibility testing

Antibiotic resistance profiles of *C*. *jejuni* and *C*. *coli* isolates were determined using the disk diffusion method as described by EUCAST (2023). Nine antimicrobial agents were tested on Mueller-Hinton agar (Pasteur Institute) supplemented with 5% sheep blood: ampicillin (10 µg), amoxicillin/clavulanic acid (20/10 µg), cefotaxime (30 µg), gentamicin (15 µg), erythromycin (15 µg), nalidixic acid (30 µg), ciprofloxacin (5 µg), tetracycline (30 µg), and chloramphenicol (30 µg). Resistance was interpreted using CASFM clinical breakpoints (2023). Multidrug resistance (MDR) was defined as resistance to three or more antibiotic classes.

### Statistical analysis

The data were analyzed using SPSS version 26.0 (IBM). The prevalence of *Campylobacter *was determined by conducting simple proportion calculations. Z-tests were used to assess the distribution of *Campylobacter* across different sample types by comparing observed proportions*. *Logistic regression models were employed to analyze the association between* Campylobacter* detection rates among workers, sample type, and other contextual factors. Statistical significance was defined as *p* < 0.05.

## Results

### Prevalence of Campylobacter spp. in different sample types

The overall prevalence of *Campylobacter* spp. across all poultry samples was 66.3% (199/300). Among poultry samples, cecal content exhibited the highest contamination rate (96%), followed by cloacal swabs (70%) and neck skin (33 %), Table 1. For slaughterhouse workers, logistic regression analysis indicated a *Campylobacter* spp. rate of 1.1% in workers from industrial slaughterhouses, compared to 3.9% in those from semi-industrial slaughterhouses. However, the difference was not statistically significant (OR = 0.284, 95% CI: 0.031–2.613, *p* = 0.266). Additionally, hand scrapings had a higher rate of detection of *Campylobacter* spp. (5.9%) compared to fecal samples (3.9%), but the difference was not statistically significant (OR = 1.547, 95% CI: 0.247–9.623, *p* = 0.644) (Table 2).

### Identification of Campylobacter jejuni and C. coli in sample types

*Campylobacter jejuni* was the predominant species, detected in 29% (87/300) of all samples, with higher prevalence in cecal content 42.5% (37/100) and cloacal swabs 40.2% (35/100) compared to neck skin 17.2% (15/100, *p* < 0.05). *Campylobacter* *coli* accounted for 11.3% (34/300) of isolates, primarily in cecal content 41.2% (14/100) and cloacal swabs 38.2% (13/100). Co-detection of *C*. *jejuni* and *C*. *coli* (CJ+CC) occurred in 4.7% (14/300) of cases, while mixed infections with other species (CJ+CT) were observed in 7.3% (22/300). Indeterminate *Campylobacter* species, which could not be fully characterized, constituted 32% (96/300) of isolates, predominantly from neck skin samples 69.8% (67/100, *p* < 0.05). All isolates from slaughterhouse workers were confirmed as *C*.* jejuni* (Table 3).

### Antimicrobial susceptibility patterns

Table 4 shows the antimicrobial resistance profile of a total of 170 isolates (*C*. *jejuni*: 115 and *C*. *coli*: 55). *Campylobacter* *jejuni* isolates exhibited high resistance to ampicillin (82.6%), nalidixic acid (49.6%), and erythromycin (33%), whereas *C*. *coli* showed resistance to ciprofloxacin (50%), tetracycline (38.9%), and nalidixic acid (40.7%). Both species remained highly susceptible to chloramphenicol (*C*. *jejuni*: 87%, *C*. *coli*: 92%) and cefotaxime (*C*.* jejuni*: 76.5%, *C*. *coli*: 87%). *Campylobacter* *jejuni* isolates from workers demonstrated complete susceptibility to tetracycline (100%). It was observed that gentamicin, erythromycin, and cefotaxime exhibited moderate resistance, with percentages of 40%, while ampicillin, amoxicillin-clavulanic acid, ciprofloxacin, nalidixic acid, and chloramphenicol demonstrated resistance rates of 25%. (Table 4).

**Table 1.** Occurrence of *Campylobacter *spp. in different sample types from poultry slaughterhouses.

Sample type	Total samples examined	Positive *Campylobacter* spp. n (%)
Cloacal swabs	100	70 (%)
Cecal content	100	96 (%)
Neck Skin (Rinsing)	100	33 (%)
Overall prevalence	300	199 (66.3%)

**Table 2.** Detection rate of *Campylobacter *spp. in slaughterhouse personnel (logistic regression results).

Factor	Coefficient (B)	OR [Exp(B)]	95% CI lower	95% CI upper	*p*-value	Detection rate % (*n*)
Sample Type						
Fecal Droppings (FD)	0	1	−	−	−	3.9 (2)
Hand Scrapings (HS)	0.432	1.541	0.247	9.623	0.644	5.9 (3)
Slaughterhouse Type						
Semi-Industrial (S)	0	1	−	−	−	3.9 (4)
Industrial (I)	−1.259	0.284	0.031	2.613	0.266	1.1 (1)
Constant	−3.070	0.046	−	−	0.000	3.9

**Table 3.** Identification of *Campylobacter jejuni* and *Campylobacter coli* in different samples.

*Campylobacter* Species	Cloacal swabs (*n* = 100)	Cecal content (*n* = 100)	Neck skin (*n* = 100)	Total (*n* = 300)	*p*-value (Z-Test)
*Campylobacter* *jejuni (CJ)*	35 (40.2%)	37 (42.5%)	15 (17.2%)	87 (29%)	< 0.05
*Campylobacter* *coli (CC)*	13 (38.2%)	14 (41.2%)	7 (20.6%)	34 (11.3%)	< 0.05
*Campylobacter jejuni + C*. *coli (CJ+CC)*	4 (28.6%)	8 (57.1%)	2 (14.3%)	14 (4.7%)	NS
*Campylobacter jejuni* + others *(CJ+CT)*	9 (40.9%)	13 (59.1%)	0 (0%)	22 (7.3%)	< 0.05
Other combinations	1 (50%)	2 (50%)	1 (0%)	4 (1.3%)	NS
Indeterminate *Campylobacter*	25 (26%)	4 (4.2%)	67 (69.8%)	96 (32%)	< 0.05
Total	100	100	100	300	-

NS: no significant.

**Table 4.** *In vitro* antimicrobial sensitivity patterns of *Campylobacter jejuni* and *Campylobacter coli* isolates from slaughterhouse in broiler chickens and workers’ isolates.

	Broiler chickens				Workers	
Isolates	*Campylobacter jejuni*		*Campylobacter coli*		*Campylobacter jejuni*	
Antibiotic	Susceptible % (*n*)	Resistant % (*n*)	Susceptible % (*n*)	Resistant % (*n*)	Susceptible % (*n*)	Resistant % (*n*)
Ampicillin (AM-10)	17.4 (20)	82.6 (95)	51.9 (29)	48.1 (26)	75 (4)	25 (1)
Amoxicillin + Clavulanic Acid (AMC)	54.8 (63)	45.2 (52)	75.9 (42)	24.1 (13)	75 (4)	25 (1)
Gentamicin (GM-15)	69.4 (80)	22.5 (26)	85.2 (47)	11.1 (6)	60 (3)	40 (2)
Erythromycin (E-15)	67.0 (77)	33.0 (38)	66.7 (37)	33.3 (18)	60 (3)	40 (2)
Tetracycline (TET-30)	65.2 (75)	34.8 (40)	59.3 (33)	38.9 (21)	100 (5)	0 (0)
Ciprofloxacin (CIP-5)	74.8 (86)	25.2 (29)	50.0 (27)	50.0 (27)	75 (4)	25 (1)
Nalidixic Acid (NA-30)	50.4 (58)	49.6 (57)	59.3 (33)	40.7 (22)	75 (4)	25 (1)
Chloramphenicol (C-30)	87.0 (100)	13.0 (15)	92.6 (51)	7.4 (4)	75 (4)	25 (1)
Cefotaxime (CTX-30)	76.5 (88)	23.5 (27)	87.0 (48)	11.1 (6)	60 (3)	40 (2)

### Multidrug resistance (MDR) profiles

**Figure 1.**
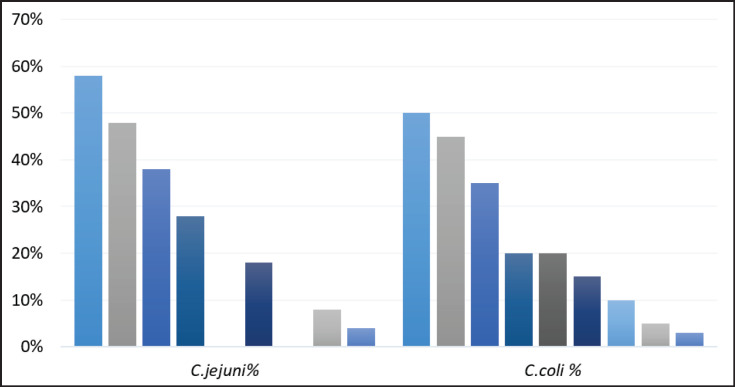
Prevalence of AMR phenotypes within multi-drug resistant (MDR: ≥ 3 AMR) isolates of *Campylobacter jejuni* and *Campylobacter coli* isolates. Ampicillin (AM); Amoxicillin + Clavulanic Acid (AMC); Gentamicin (GM); Erythromycin (E); Tetracycline (TET); Ciprofloxacin (CIP); Nalidixic Acid (NA); Chloramphenicol (C); Cefotaxime (CTX).

Isolates were considered MDR if they had 3 or more AMR phenotypes. The analysis of multidrug resistance patterns of strains (88/170) revealed significant differences between *C*.* jejuni* (62/115) and *C*. *coli* (26/55), particularly regarding resistance to critical antibiotics. *Campylobacter jejuni* exhibited higher multidrug resistance levels, particularly in combinations involving ciprofloxacin 58% (36/62), tetracycline 48% (30/62), and erythromycin 38% (24/62) (Fig. 1). While *C*.* coli* displayed lower MDR, notable resistance was observed for ciprofloxacin 50% (13/26), tetracycline 45% (12/26), and erythromycin 35% (9/26).

## Discussion

In the current study, it was determined that the prevalence of *Campylobacter* positivity varies depending on the sample types in the same slaughterhouse, indicating that sample type changes the detection rate of *Campylobacter* spp. in poultry. The higher prevalence in cecal content and cloacal swabs compared to neck skin may be attributed to differences in contamination levels or detection efficiency. Contamination levels obtained in the current study align with previous findings reported by Baali et al. [20], who also found an overall prevalence of *Campylobacter* spp. in slaughterhouses of 62.5%, with 70% in cecal contents, 65% in cloacal swabs, and 55% in neck skin, in the same region. In contrast, studies conducted in central Algeria by Messad [20] and Bouhamed et al. [25] reported higher prevalence rates: 73%–98% in cecal contents and 75%–80% in neck skin samples, respectively. The regional disparity is likely influenced by differences in slaughter practices: industrial facilities in eastern Algeria employ stricter hygiene protocols, whereas in traditional and semi-industrial slaughterhouses, which are prevalent in central regions, manual handling increases contamination risks. Detection methods (culture *vs.* molecular) and sample type also influence prevalence, as evidenced by studies from Morocco, Tunisia, and Egypt [26–28].

Although a higher rate of contamination was observed among workers in semi-industrial facilities compared with those in industrial facilities, the difference was not statistically significant (OR = 0.284; *p* = 0.266).

However, this pattern is consistent with findings from South African semi-industrial settings, in contrast with European high-tech slaughterhouses, where worker contamination is rare [29]. Hand swabs from workers showed a higher (though not statistically significant) detection rate (5.9%) compared to fecal samples (3.9%) (OR = 1.541, *p* = 0.644), emphasizing the role of direct contact in occupational exposure. Although *Campylobacter* spp. was detected among slaughterhouse personnel, the overall prevalence remained relatively low. Further investigation into transmission routes is needed to confirm the possibility of a correlation between the two types of samples (fecal and hand samples), and hygiene compliance is warranted to reduce both workers’ and public health risks.

Analysis of *Campylobacter* species distribution across sample types revealed relevant data. *Campylobacter jejuni* was more prevalent (29%) than *C*. *coli* (11.3%), with higher detection rates in cecal contents (42.5%) and cloacal swabs (40.2%), as confirmed by the Z-test. In Ethiopia, Belina et al. [30] reported *Campylobacter* prevalence up to 44%, slightly higher than the present findings, likely reflecting differences in hygiene and processing practices. Several studies from developed countries confirm that the caeca serve as the primary colonization site for *Campylobacter* in poultry. For instance, Weerasooriya et al. [31] reported similar strain distribution in avian matrices, and Adhikari et al. [32] found elevated intestinal colonization by *C*. *jejuni*, influenced by rearing conditions. The latter study further confirmed that caeca act as the main reservoir in antibiotic-free poultry farms in the United States. Al Hakeem et al. [33] emphasized strain variability depending on environmental factors and production systems, potentially explaining inter-study differences.

While *C*. *jejuni* predominates, both species can co-exist in avian hosts, and their interaction within the gut microbiota is influenced by environmental conditions and production practices [31]. Co-occurrence is more frequent in high-density farms, where contamination pressure is elevated [32]. *Campylobacter coli* demonstrates greater resistance to environmental stress in antibiotic-free production systems, favoring its persistence [32]. Furthermore, *C*. *jejuni* and *C*. *coli* may exchange genetic material, including AMR genes, enhancing adaptation to farming and processing environments [33].

In the current study, *C*. *jejuni* exhibited high rates of resistance to ciprofloxacin (74.8%), ampicillin (82.6%), and tetracycline (34.8%). *Campylobacter* *coli* showed resistance to ciprofloxacin (50%) and tetracycline (38.9%), consistent with trends reported in Tunisia and Morocco [28,34,35]. The detection of plasmid-mediated quinolone resistance genes and novel sequence types (e.g., ST13450) in Tunisian *C*. *coli* isolates [35] highlights the role of horizontal gene transfer in AMR dissemination. MDR was prominent in *C*. *jejuni* 53.9% (62/115), particularly to ciprofloxacin-tetracycline-erythromycin combinations 38.7% (24/62), aligning with findings from Tunisia and the United States [36,37]. The increased resistance observed in *C*. *jejuni* compared with *C*. *coli* could result from selection pressure exerted by the intensive use of antibiotics in poultry farming, the primary source of *C*. *jejuni* isolates in our study. This phenomenon could also be influenced by local circulation of resistant clones, and differences in the distribution of isolates between species may also contribute to this atypical pattern. Targeted molecular analysis is needed to elucidate the determining factors. These patterns correlate with unregulated antibiotic use in Algerian poultry production, which promotes resistance and further compromises therapeutic efficacy in humans.

On a global scale, AMR in *Campylobacter* spp. represents a growing public health threat. In Europe, ciprofloxacin resistance exceeds 60% in several countries [38]. Similar trends have been observed in Africa, where unregulated use of antibiotics in the poultry industry has driven the emergence of resistant strains [39]. In Asia, MDR rates of up to 60% reflect the pressure of intensive farming [40]. These converging trends highlight the global consequences of unregulated antibiotic use and intensive livestock production. 

## Conclusion

This study demonstrates a high prevalence of thermotolerant *Campylobacter* spp. in Algerian poultry slaughterhouses, with *C*. *jejuni* dominating isolates and exhibiting alarming resistance to ciprofloxacin, tetracycline, and multidrug resistance. The disparity in contamination rates between industrial and semi-industrial facilities underscores the impact of slaughterhouse hygiene practices, while the detection of *C*.* jejuni* in slaughterhouse workers highlights occupational zoonotic risks. Notably, the near-ubiquitous resistance to ampicillin and emerging resistance to erythromycin and gentamicin reflect systemic antibiotic misuse in poultry production, mirroring trends across North Africa. These findings necessitate urgent, multidisciplinary action under the One Health framework. Prioritizing antibiotic stewardship in agriculture, enhancing biosecurity protocols in slaughterhouses, and adopting alternatives such as bacteriophage therapy or vaccines are critical to curbing resistance gene dissemination. Furthermore, regional genomic surveillance programs must be established to monitor resistance dynamics, while international collaboration is imperative to standardize AMR mitigation policies. By integrating these measures, Algeria can mitigate the escalating threat of untreatable *Campylobacter* infections, safeguarding both public health and food security in an era of rising antimicrobial resistance.

## List of abbreviations

AMR, Antimicrobial resistance; MDR, Multidrug resistance; *C. Jejuni*, *Campylobacter jejuni*; *C. coli*, *Campylobacter coli*; AM, ampicillin; AMC, amoxicillin/clavulanic acid; CTX, cefotaxime; GM, gentamicin; E, erythromycin; NA, nalidixic acid; CIP, ciprofloxacin; TET, tetracycline; C, chloramphenicol; h, hour.
